# Supplementary material for: Circulating mucosal-like IgA responses increase with severity of Puumala orthohantavirus-caused hemorrhagic fever with renal syndrome
Source: Front Immunol. 2024 Oct 24;15:1480041. doi: 10.3389/fimmu.2024.1480041 (PMC11540702; doi:10.3389/fimmu.2024.1480041)
Supplement: Supplementary file 1 [file DataSheet1.pdf]

Results of Generalized estimating equations (GEE)

Parameter estimates of GEE modeling of the indicated dependent variable across days post onset of fever and calculating significant differences as compared to day 360, representing full recovery. GEE was computed by SPSS setting working correlation matrix to independent and the model type to gamma with log link. The models are based on data points presented in Figs 1 and 3 as well as supplementary Fig. 2.

a. Set to zero because this parameter is redundant.

			95% Wald Confidence Interval		Hypothesis Test		
Parameter	B	Std. Error	Lower	Upper	Wald Chi-Square	df	Sig.
(Intercept)	-3,131	,3516	-3,820	-2,441	79,265	1	<,001
[day=6,00]	3,013	,4156	2,199	3,828	52,561	1	<,001
[day=7,00]	2,425	,4453	1,552	3,297	29,654	1	<,001
[day=8,00]	2,555	,4073	1,756	3,353	39,332	1	<,001
[day=9,00]	2,160	,4086	1,359	2,961	27,941	1	<,001
[day=30,00]	,511	,4231	-,318	1,340	1,460	1	,227
[day=180,00]	-,493	,3743	-1,227	,240	1,737	1	,187
[day=360,00]	0[a]	.	.	.	.	.	.
(Scale)	1,279						

Dependent Variable: frequency of PBs in all single cells

			95% Wald Confidence Interval		Hypothesis Test		
Parameter	B	Std. Error	Lower	Upper	Wald Chi-Square	df	Sig.
(Intercept)	-1,684	,1595	-1,997	-1,372	111,515	1	<,001
[day=6,00]	-1,096	,2763	-1,637	-,554	15,726	1	<,001
[day=7,00]	-,651	,2844	-1,208	-,094	5,238	1	,022
[day=8,00]	-,704	,2305	-1,156	-,252	9,334	1	,002
[day=9,00]	-1,487	,2250	-1,928	-1,046	43,672	1	<,001
[day=30,00]	-,650	,1876	-1,018	-,282	11,995	1	<,001
[day=180,00]	-,273	,1723	-,611	,064	2,515	1	,113
[day=360,00]	0[a]	.	.	.	.	.	.
(Scale)	,638						

Dependent Variable: Frequency of CCR9 in PBs

Parameter	B	Std. Error	95% Wald Confidence Interval Lower	Upper	Hypothesis Test Wald Chi-Square	df	Sig.
(Intercept)	-,723	,1020	-,923	-,523	50,241	1	<,001
[day=6,00]	-,496	,1512	-,793	-,200	10,783	1	,001
[day=7,00]	-,084	,1323	-,343	,176	,399	1	,528
[day=8,00]	-,121	,1541	-,423	,181	,617	1	,432
[day=9,00]	-,124	,1945	-,506	,257	,409	1	,522
[day=30,00]	-,132	,1120	-,352	,087	1,398	1	,237
[day=180,00]	-,226	,0933	-,409	-,043	5,845	1	,016
[day=360,00]	0[a]	.	.	.	.	.	.
(Scale)	,286						

Dependent Variable: Frequency of a4b7 in PBs

Parameter	B	Std. Error	95% Wald Confidence Interval Lower	Upper	Hypothesis Test Wald Chi-Square	df	Sig.
(Intercept)	-,431	,0691	-,567	-,296	39,002	1	<,001
[day=6,00]	,265	,0684	,131	,399	15,011	1	<,001
[day=7,00]	,245	,0525	,142	,347	21,725	1	<,001
[day=8,00]	,236	,0840	,071	,400	7,896	1	,005
[day=9,00]	,256	,0662	,126	,386	14,945	1	<,001
[day=30,00]	,111	,0652	-,017	,238	2,880	1	,090
[day=180,00]	,011	,0773	-,141	,162	,019	1	,889
[day=360,00]	0[a]	.	.	.	.	.	.
(Scale)	,064						

Dependent Variable: Frequency of CD11b in PBs

Parameter	B	Std. Error	95% Wald Confidence Interval		Hypothesis Test		
			Lower	Upper	Wald Chi-Square	df	Sig.
(Intercept)	-2,142	,1497	-2,436	-1,849	204,706	1	<,001
[day=6,00]	,278	,2033	-,120	,676	1,870	1	,172
[day=7,00]	,140	,2054	-,263	,542	,463	1	,496
[day=8,00]	,085	,1948	-,297	,467	,191	1	,662
[day=9,00]	,015	,1472	-,274	,303	,010	1	,920
[day=30,00]	-,144	,1797	-,496	,208	,646	1	,422
[day=180,00]	-,046	,1221	-,285	,194	,141	1	,708
[day=360,00]	0[a]	.	.	.	.	.	.
(Scale)	,415						

Dependent Variable: Frequency of B1 in all single cells

Parameter	B	Std. Error	95% Wald Confidence Interval		Hypothesis Test		
			Lower	Upper	Wald Chi-Square	df	Sig.
(Intercept)	-2,193	,1483	-2,484	-1,903	218,826	1	<,001
[day=6,00]	,309	,2069	-,096	,714	2,231	1	,135
[day=7,00]	,167	,2093	-,243	,577	,635	1	,425
[day=8,00]	,106	,1977	-,282	,493	,287	1	,592
[day=9,00]	-,038	,1749	-,381	,304	,048	1	,826
[day=30,00]	-,180	,2087	-,589	,229	,742	1	,389
[day=180,00]	-,010	,1328	-,270	,250	,006	1	,940
[day=360,00]	0[a]	.	.	.	.	.	.
(Scale)	,432						

Dependent Variable: Frequency of B1 in all single cells

			95% Wald Confidence Interval		Hypothesis Test		
Parameter	B	Std. Error	Lower	Upper	Wald Chi-Square	df	Sig.
(Intercept)	-3,593	,1054	-3,800	-3,387	1162,306	1	<,001
[day=6,00]	,587	,1561	,281	,893	14,134	1	<,001
[day=7,00]	,392	,1291	,139	,645	9,225	1	,002
[day=8,00]	,440	,2333	-,017	,897	3,557	1	,059
[day=9,00]	,366	,2829	-,188	,921	1,676	1	,195
[day=30,00]	,633	,1552	,328	,937	16,613	1	<,001
[day=180,00]	,192	,1592	-,120	,504	1,453	1	,228
[day=360,00]	0[a]	.	.	.	.	.	.
(Scale)	,443						

Dependent Variable: Frequency of IgA in B1 cells

			95% Wald Confidence Interval		Hypothesis Test		
Parameter	B	Std. Error	Lower	Upper	Wald Chi-Square	df	Sig.
(Intercept)	-1,175	,2014	-1,570	-,781	34,067	1	<,001
[day=6,00]	,020	,2242	-,419	,460	,008	1	,928
[day=7,00]	-,154	,2351	-,614	,307	,427	1	,513
[day=8,00]	-,336	,2104	-,748	,076	2,553	1	,110
[day=9,00]	-,572	,2276	-1,019	-,126	6,324	1	,012
[day=30,00]	-,186	,2347	-,646	,274	,627	1	,428
[day=180,00]	,340	,2209	-,093	,773	2,363	1	,124
[day=360,00]	0[a]	.	.	.	.	.	.
(Scale)	,280						

Dependent Variable: Frequency of IgA in PB

			95% Wald Confidence Interval		Hypothesis Test		
Parameter	B	Std. Error	Lower	Upper	Wald Chi-Square	df	Sig.
(Intercept)	-,494	,0774	-,646	-,342	40,687	1	<,001
[day=6,00]	-,316	,1331	-,577	-,055	5,640	1	,018
[day=7,00]	-,108	,1149	-,333	,118	,876	1	,349
[day=8,00]	-,098	,1200	-,334	,137	,673	1	,412
[day=9,00]	-,112	,1151	-,338	,113	,951	1	,329
[day=30,00]	-,164	,1300	-,419	,091	1,586	1	,208
[day=180,00]	-,319	,1091	-,533	-,105	8,539	1	,003
[day=360,00]	0[a]	.	.	.	.	.	.
(Scale)	,215						

Dependent Variable: The frequency of a4b7 in IgA PBs

			95% Wald Confidence Interval		Hypothesis Test		
Parameter	B	Std. Error	Lower	Upper	Wald Chi-Square	df	Sig.
(Intercept)	-,416	,0749	-,562	-,269	30,832	1	<,001
[day=6,00]	,226	,0751	,078	,373	9,018	1	,003
[day=7,00]	,183	,0543	,076	,289	11,295	1	<,001
[day=8,00]	,147	,0915	-,032	,327	2,584	1	,108
[day=9,00]	,162	,0850	-,004	,329	3,641	1	,056
[day=30,00]	,009	,0768	-,141	,160	,014	1	,905
[day=180,00]	-,077	,1179	-,308	,154	,424	1	,515
[day=360,00]	0[a]	.	.	.	.	.	.
(Scale)	,081						

Dependent Variable: Frequency of CD11b in IgA PBs

			95% Wald Confidence Interval		Hypothesis Test		
Parameter	B	Std. Error	Lower	Upper	Wald Chi-Square	df	Sig.
(Intercept)	-1,796	,1222	-2,035	-1,556	215,992	1	<,001
[day=6,00]	-,208	,2177	-,635	,219	,913	1	,339
[day=7,00]	-,038	,1817	-,394	,318	,045	1	,833
[day=8,00]	-,073	,1778	-,422	,275	,169	1	,681
[day=9,00]	-,291	,1896	-,663	,080	2,359	1	,125
[day=30,00]	,368	,1314	,110	,625	7,833	1	,005
[day=180,00]	,200	,1267	-,048	,449	2,498	1	,114
[day=360,00]	0^a	.	.	.	.	.	.
(Scale)	,326						

Dependent Variable: Frequency of CCR9 in all B1

			95% Wald Confidence Interval		Hypothesis Test		
Parameter	B	Std. Error	Lower	Upper	Wald Chi-Square	df	Sig.
(Intercept)	-,675	,0804	-,832	-,517	70,323	1	<,001
[day=6,00]	-,235	,1086	-,448	-,022	4,690	1	,030
[day=7,00]	,008	,0726	-,135	,150	,011	1	,917
[day=8,00]	-,111	,0823	-,272	,050	1,827	1	,177
[day=9,00]	-,019	,0960	-,207	,169	,038	1	,845
[day=30,00]	,039	,0690	-,096	,175	,325	1	,569
[day=180,00]	-,047	,0645	-,173	,079	,530	1	,467
[day=360,00]	0^a	.	.	.	.	.	.
(Scale)	,111						

Dependent Variable: Frequency of a4b7 in B1

Parameter	B	Std. Error	95% Wald Confidence Interval		Hypothesis Test		
			Lower	Upper	Wald Chi-Square	df	Sig.
(Intercept)	-,639	,0548	-,747	-,532	136,277	1	<,001
[day=6,00]	-,062	,0755	-,210	,086	,675	1	,411
[day=7,00]	-,062	,0610	-,181	,058	1,021	1	,312
[day=8,00]	-,141	,0690	-,276	-,005	4,149	1	,042
[day=9,00]	-,145	,0849	-,311	,021	2,913	1	,088
[day=30,00]	-,063	,0479	-,157	,031	1,724	1	,189
[day=180,00]	-,052	,0780	-,205	,101	,450	1	,502
[day=360,00]	0[a]	.	.	.	.	.	.
(Scale)	,065						

Dependent Variable: Frequency of CD11b in B1

Parameter	B	Std. Error	95% Wald Confidence Interval		Hypothesis Test		
			Lower	Upper	Wald Chi-Square	df	Sig.
(Intercept)	2,385	,1494	2,093	2,678	255,039	1	<,001
[day=5,00]	,009	,2055	-,394	,411	,002	1	,966
[day=6,00]	-,088	,1701	-,422	,245	,270	1	,603
[day=7,00]	-,022	,1790	-,373	,329	,015	1	,902
[day=8,00]	-,191	,1853	-,554	,173	1,057	1	,304
[day=9,00]	,180	,2252	-,262	,621	,636	1	,425
[day=30,00]	,118	,1557	-,187	,423	,572	1	,450
[day=180,00]	-,139	,1586	-,450	,172	,764	1	,382
[day=360,00]	0[a]	.	.	.	.	.	.
(Scale)	,406						

Dependent Variable: total IgA in serum

			95% Wald Confidence Interval		Hypothesis Test		
Parameter	B	Std. Error	Lower	Upper	Wald Chi-Square	df	Sig.
(Intercept)	-1,572	,1472	-1,861	-1,284	114,093	1	<,001
[day=5,00]	1,224	,1827	,866	1,582	44,926	1	<,001
[day=6,00]	1,255	,1473	,966	1,544	72,566	1	<,001
[day=7,00]	1,289	,1612	,973	1,605	63,954	1	<,001
[day=8,00]	1,310	,1525	1,011	1,609	73,763	1	<,001
[day=9,00]	1,181	,1531	,881	1,481	59,542	1	<,001
[day=30,00]	1,047	,1486	,756	1,338	49,697	1	<,001
[day=180,00]	-,130	,1407	-,406	,145	,857	1	,355
[day=360,00]	0[a]	.	.	.	.	.	.
(Scale)	,383						

Dependent Variable: N-IgA in serum

			95% Wald Confidence Interval		Hypothesis Test		
Parameter	B	Std. Error	Lower	Upper	Wald Chi-Square	df	Sig.
(Intercept)	-2,086	,0248	-2,135	-2,037	7054,810	1	<,001
[day=5,00]	,760	,1273	,511	1,009	35,675	1	<,001
[day=6,00]	,849	,1063	,641	1,057	63,822	1	<,001
[day=7,00]	,758	,0922	,578	,939	67,613	1	<,001
[day=8,00]	,532	,0549	,424	,639	93,712	1	<,001
[day=9,00]	,470	,0646	,343	,597	52,908	1	<,001
[day=30,00]	,243	,0321	,180	,305	57,178	1	<,001
[day=180,00]	-,009	,0153	-,039	,021	,370	1	,543
[day=360,00]	0[a]	.	.	.	.	.	.
(Scale)	,126						

Dependent Variable: DNP-IgA in serum

			95% Wald Confidence Interval		Hypothesis Test		
Parameter	B	Std. Error	Lower	Upper	Wald Chi-Square	df	Sig.
(Intercept)	-2,133	,1942	-2,513	-1,752	120,610	1	<,001
[day=5,00]	,700	,3317	,050	1,350	4,451	1	,035
[day=6,00]	,675	,2197	,244	1,106	9,442	1	,002
[day=7,00]	,754	,2370	,289	1,218	10,113	1	,001
[day=8,00]	,505	,2061	,101	,909	5,991	1	,014
[day=9,00]	,756	,2154	,334	1,179	12,329	1	<,001
[day=30,00]	,325	,1860	-,039	,690	3,062	1	,080
[day=180,00]	-,420	,1944	-,801	-,039	4,663	1	,031
[day=360,00]	0[a]	.	.	.	.	.	.
(Scale)	,756						

Dependent Variable: sIgA in serum
