# Supplementary figures and images for: Circulating mucosal-like IgA responses increase with severity of Puumala orthohantavirus-caused hemorrhagic fever with renal syndrome
Source: Front Immunol. 2024 Oct 24;15:1480041. doi: 10.3389/fimmu.2024.1480041 (PMC11540702; doi:10.3389/fimmu.2024.1480041)

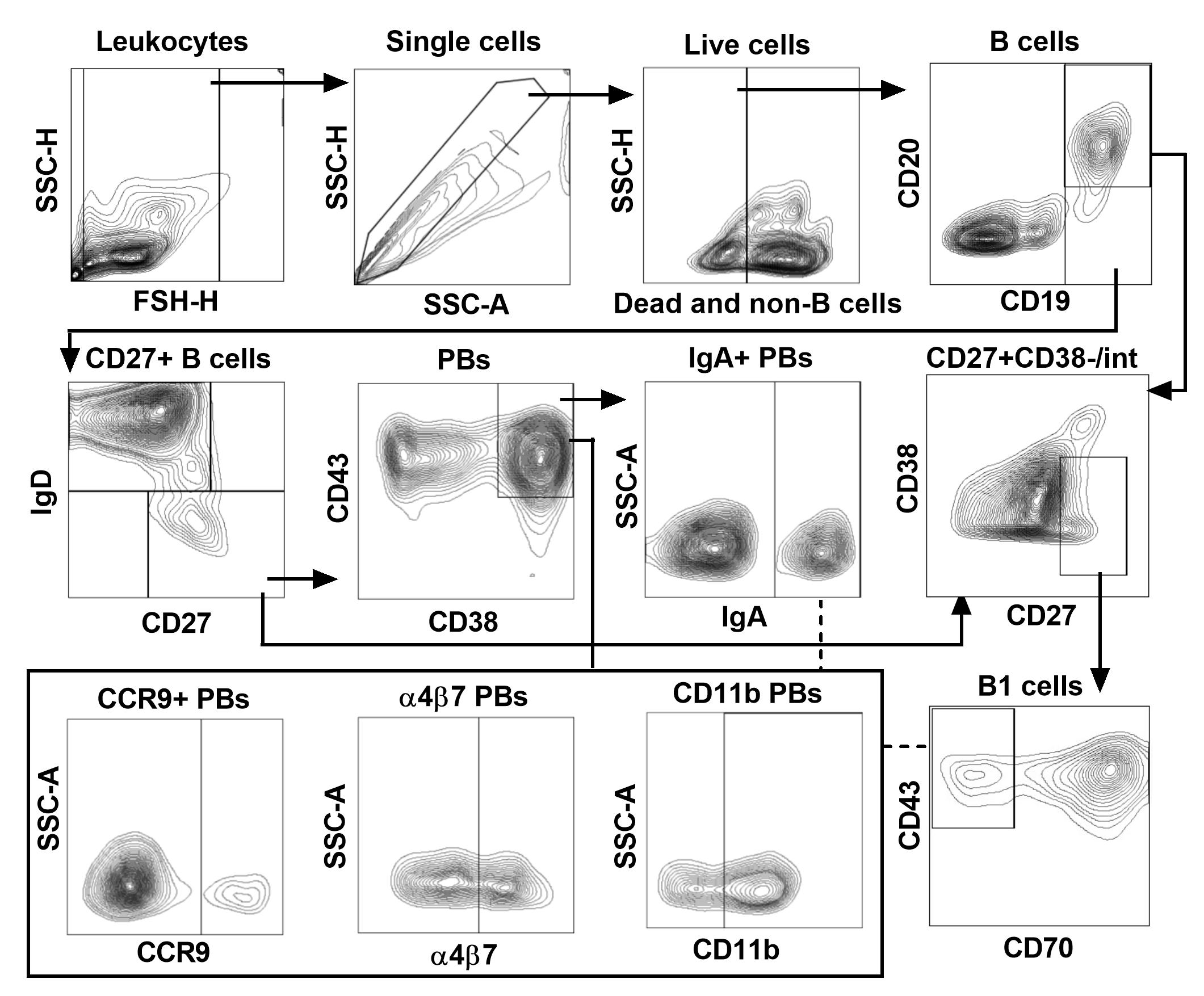

Supplement: Supplementary Figure 1 — Gating strategy allowing for the identification of PBs and B1 cells in PBMC of PUUV-HFRS patients by flow cytometry. After gating of PBMCs (SSC-H vs. FSC-H), single cells (SSC-H vs. SSC-A) and live cells (excluding also non-B cells expressing CD3, CD14, CD56 and CD66), B cells were identified as positive for either CD19, CD20 or both. PBs were identified as CD20 ± CD19+IgD-CD27+CD38++CD43+ cells and B1 cells as CD20+CD27+CD38-/intCD43+CD70-. The cells positive for surface expression of IgA, integrin α4β7, CD11b or CCR9 in PBs and B1 were gated as indicated. Contour plots are shown. [file Image1.jpeg]

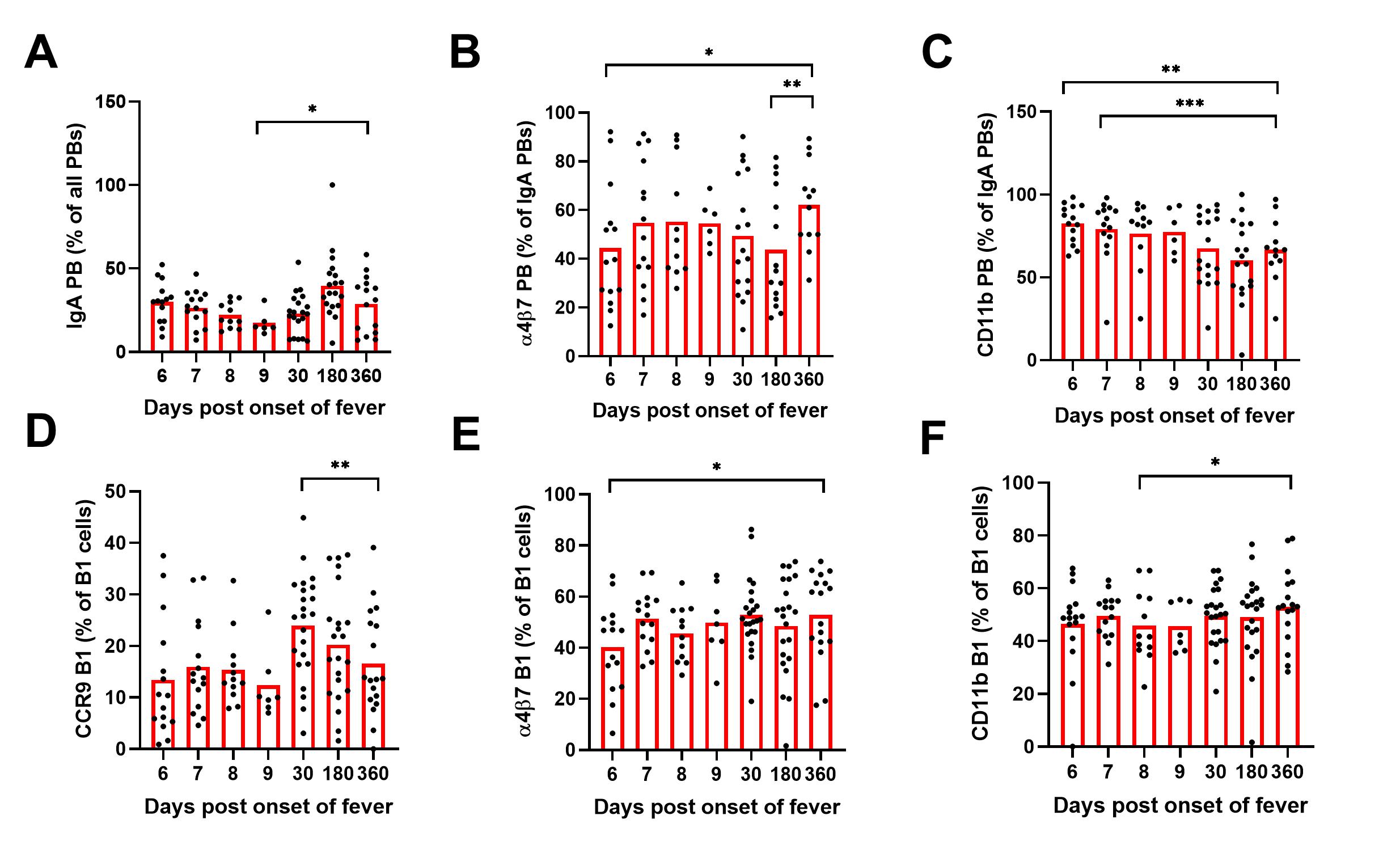

Supplement: Supplementary Figure 2 — Multiparameter flow cytometric analysis of IgA PBs and B1 cells in peripheral blood of PUUV-HFRS. PBMCs from hospitalized (days 6-9 post onset of fever, n = 14-30 per day), two weeks after discharge (~30 days post onset fever, n =23) and recovered (180 and 360 days post-onset of fever, n = 21-23) patients (total n = 25) were stained with a panel fluorochrome-labeled antibodies and live/dead green viability stain. Stained cells were analyzed by flow cytometry. (A) The frequencies of PBs expressing cell surface IgA. (B) The frequencies of IgA PBs expressing surface integrin α4β7. (C) The frequencies of IgA PBs expressing surface integrin CD11b. (D) The frequencies of B1 cells expressing surface integrin α4β7. (E) The frequencies of B1 cells expressing surface CD11b. (F) The frequencies of B1 cells expressing surface CCR9. Statistically significant differences at each time point as compared to recovery at 360 days post-onset of fever were assessed by generalized estimating equations. ***, ** and * indicate p-values <0.001, < 0.01 and < 0.05, respectively. [file Image2.jpeg]

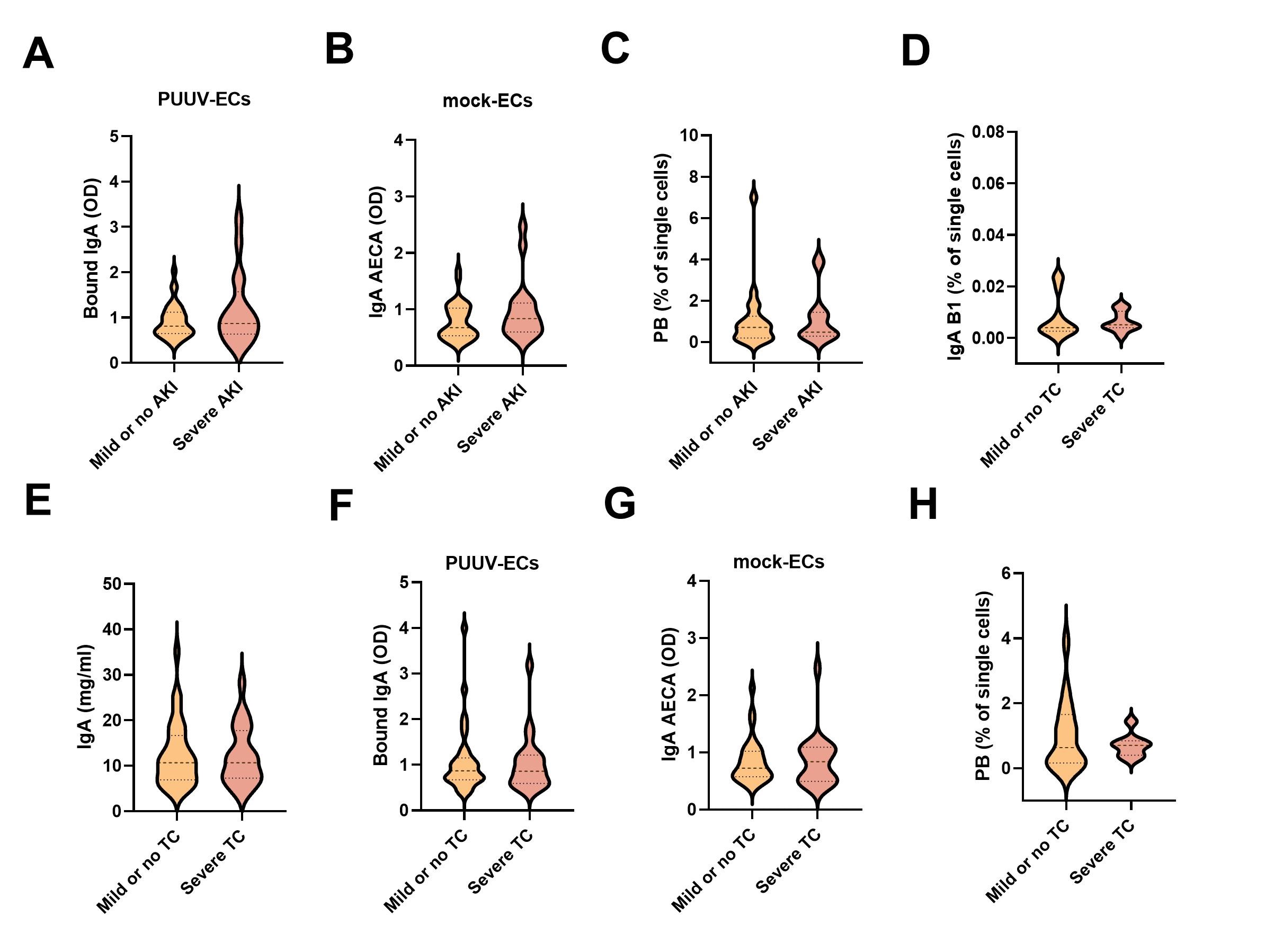

Supplement: Supplementary Figure 3 — Association between measured soluble IgA and IgA ASC levels with parameters of disease severity. (A–C) The PUUV-HFRS patients (n = 55) were stratified based the maximum blood creatinine levels measured during hospitalization as mild (blood creatinine ≤ 265 µmol/l = AKI stage 2 or lower, n = 36) or severe (blood creatinine >265 µmol/l = AKI stage 3, n = 19) or (D–H) minimum thrombocyte levels as mild or no thrombocytopenia (TC, ≥ 50 * 104/µl blood, n = 33) and severe TC (< 50 * 104/µl blood, n = 21). The maximum levels of PUUV-EC binding IgA (A, F), IgA AECA (B, G), PBs (C, F), IgA B1 cells (D) and total IgA (E) measured during hospitalization were grouped based on patient severity criteria and significant differences assessed by Mann-Whitney test. [file Image3.jpeg]

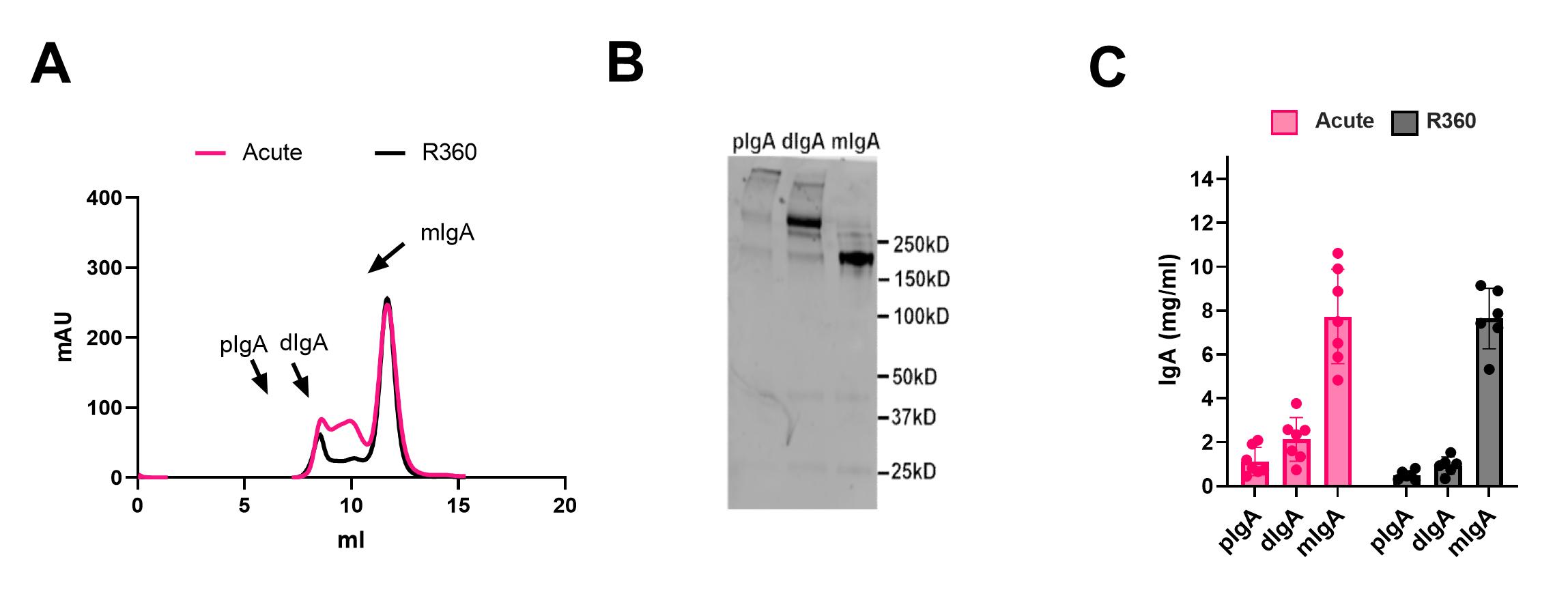

Supplement: Supplementary Figure 4 — Isolation of mIgA, dIgA and pIgA from serum of PUUV-HFRS patients. (A) Representative chromatogram of the separation of pIgA, dIgA and mIgA from total IgA isolated from acute (1st day of hospitalization and recovery stage (R360) of PUUV-HFRS. (B) Representative non-reducing western blot indicating the molecular weight of isolated mIgA (~180 kDa), dIgA (~360 kDa) and pIgA (> 360 kDa) fractions. The IgA bands were visualized by a polyclonal anti-kappa light chain antibody followed by IRdye-conjugated secondary antibody. Molecular size was estimated using as molecular weight standard on separate lane. (C). The IgA concentration of isolated IgA fractions from acute (n = 7 and R360 phase (n = 6) of PUUV-HFRS were measured by total IgA ELISA. [file Image4.jpeg]

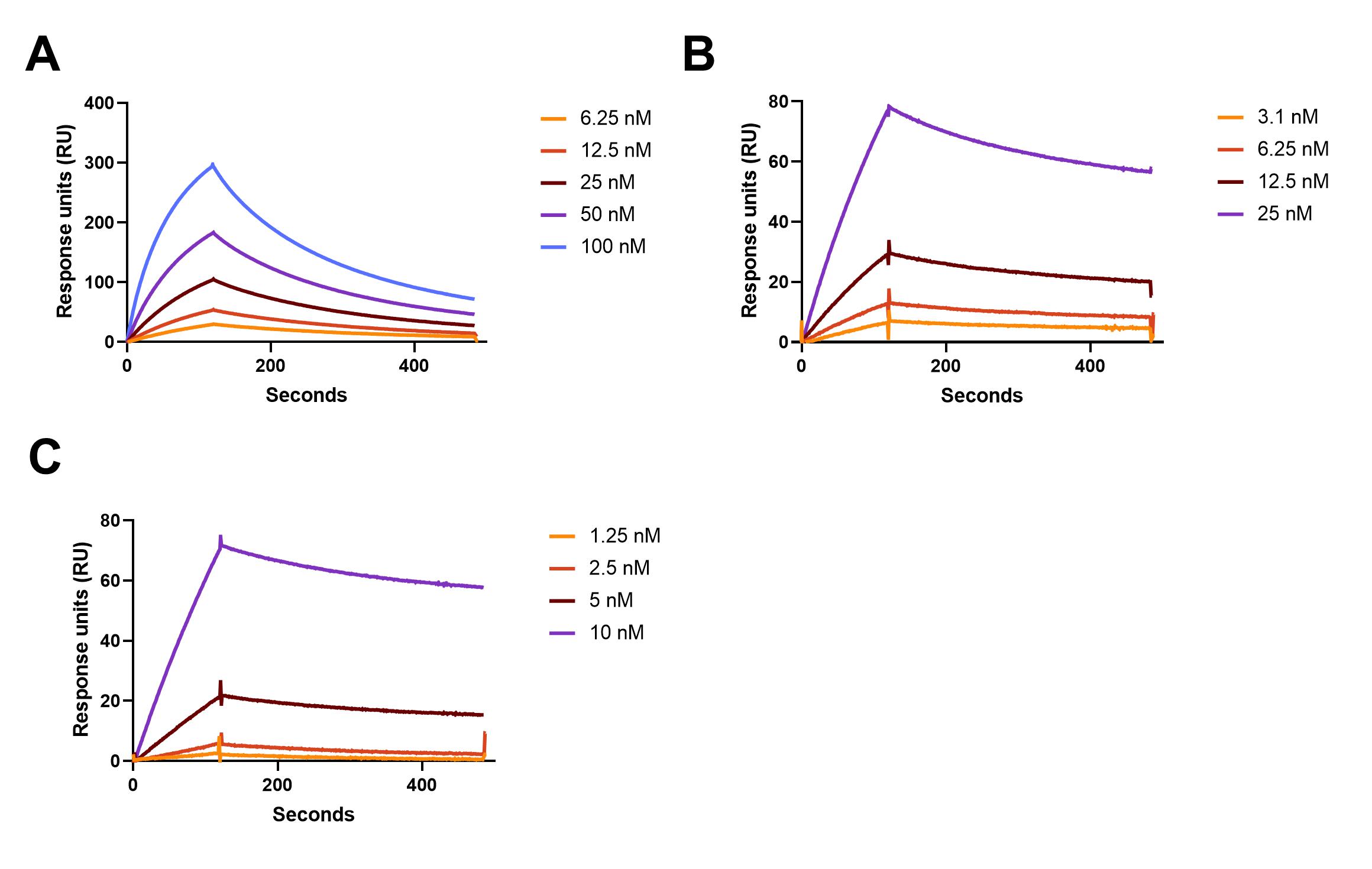

Supplement: Supplementary Figure 5 — CD89 receptor binding kinetics of circulating mIgA, dIgA and pIgA isolated from convalescent PUUV-HFRS analyzed by surface plasmon resonance. The IgA receptor CD89 was coated on the surface of biacore sensor chip and different IgA fractions, (A) mIgA, (B) dIgA and (C) pIgA, isolated from recovery PUUV-HFRS (360 days post onset of fever, n = 6), pooled at equal ratio, and used at indicated concentrations as analytes in a surface plasmon resonance binding kinetics assay using Biacore T100. [file Image5.jpeg]

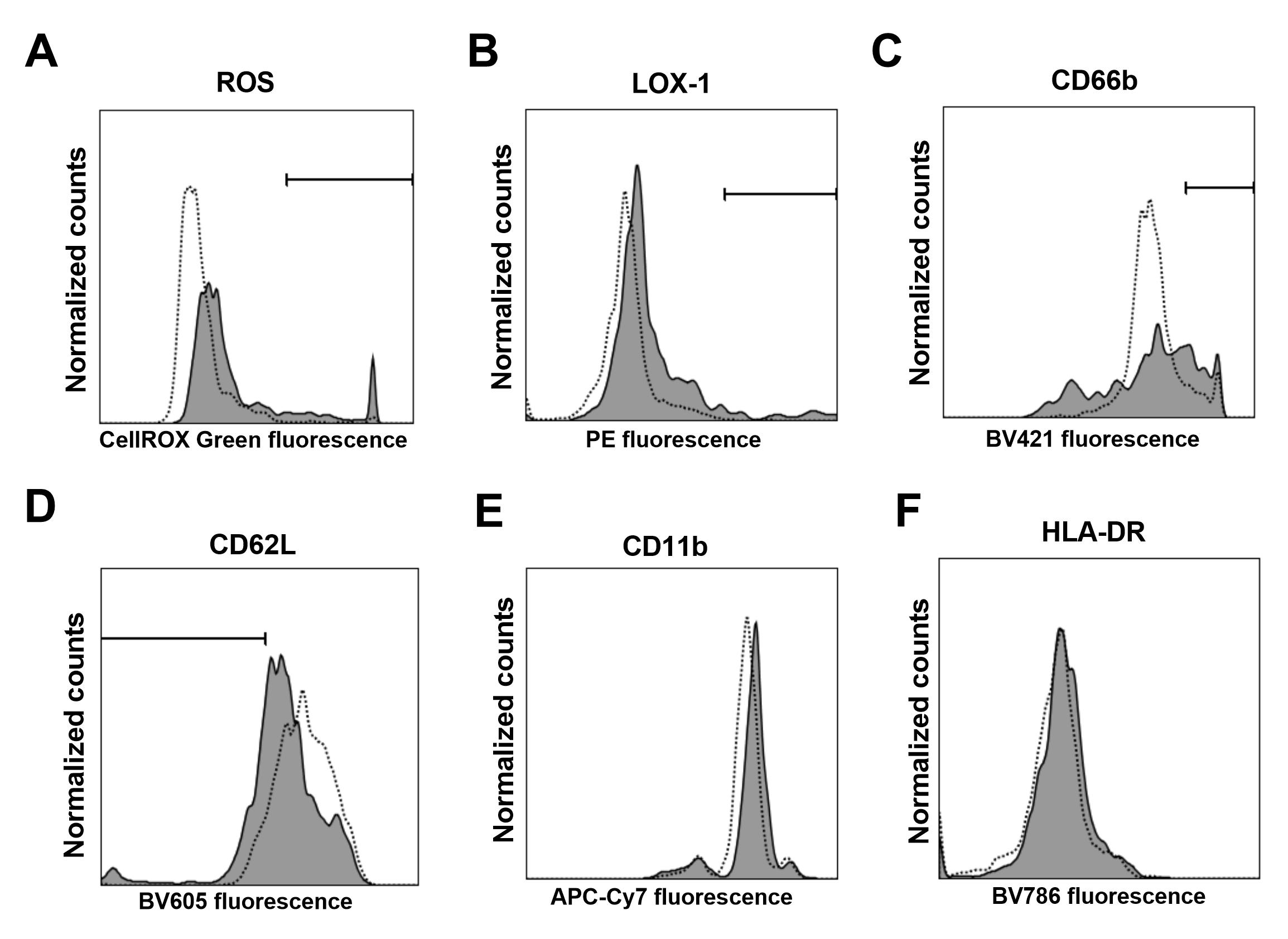

Supplement: Supplementary Figure 6 — Representative histograms of pro-inflammatory marker expression in neutrophils. Neutrophils were isolated from healthy volunteers and incubated mIgA, dIgA and pIgA isolated from PUUV-HFRS patient serum at acute (n = 7) vs. recovery stages (n = 6) of the disease. The expression of indicated markers were assessed by flow cytometry and representative histograms of pIgA (solid line with grey area)- vs. mIgA (dotted line with white area)-treated neutrophils are shown. The gating area to select ROS (A) and LOX-1 (B) positive cells as well as cells with high CD66b expression (C) is indicated. The cells with diminished CD62L expression were selected by gating as indicated in (D). The increased expression of CD11b (E) and HLA-DR (F) were assessed by Median fluorescence intensity (MFI). [file Image6.jpeg]
